# Stable preparations of tyrosine hydroxylase provide the solution structure of the full-length enzyme

**DOI:** 10.1038/srep30390

**Published:** 2016-07-27

**Authors:** Maria T. Bezem, Anne Baumann, Lars Skjærven, Romain Meyer, Petri Kursula, Aurora Martinez, Marte I. Flydal

**Affiliations:** 1 Department of Biomedicine, University of Bergen, Bergen, Norway; 2 K.G. Jebsen Centre for Neuropsychiatric Disorders, University of Bergen, Bergen, Norway; 3 Kronstad District Psychiatric Centre, Haukeland University Hospital Bergen, Norway; 4 Centre for Geobiology and Department of Earth Science, University of Bergen, Bergen, Norway and GFZ German Research Centre for Geosciences, Section 3.3, Earth Surface Geochemistry, Telegrafenberg, Potsdam, Germany; 5 Department of Neurology, Haukeland University Hospital, Bergen, Norway

## Abstract

Tyrosine hydroxylase (TH) catalyzes the rate-limiting step in the biosynthesis of catecholamine neurotransmitters. TH is a highly complex enzyme at mechanistic, structural, and regulatory levels, and the preparation of kinetically and conformationally stable enzyme for structural characterization has been challenging. Here, we report on improved protocols for purification of recombinant human TH isoform 1 (TH1), which provide large amounts of pure, stable, active TH1 with an intact N-terminus. TH1 purified through fusion with a His-tagged maltose-binding protein on amylose resin was representative of the iron-bound functional enzyme, showing high activity and stabilization by the natural feedback inhibitor dopamine. TH1 purified through fusion with a His-tagged ZZ domain on TALON is remarkably stable, as it was partially inhibited by resin-derived cobalt. This more stable enzyme preparation provided high-quality small-angle X-ray scattering (SAXS) data and reliable structural models of full-length tetrameric TH1. The SAXS-derived model reveals an elongated conformation (*D*_*max*_ = 20 nm) for TH1, different arrangement of the catalytic domains compared with the crystal structure of truncated forms, and an N-terminal region with an unstructured tail that hosts the phosphorylation sites and a separated Ala-rich helical motif that may have a role in regulation of TH by interacting with binding partners.

Tyrosine hydroxylase (TH, 1.14.16.2) catalyzes the conversion of L-tyrosine (L-Tyr) to L-3,4-dihydroxyphenylalanine (L-DOPA), which is the rate-limiting step in the synthesis of the catecholamine neurotransmitters and hormones dopamine (DA), noradrenaline, and adrenaline^1^. TH requires an enzyme-bound non-heme ferrous iron (Fe^2+^), 6*R*-tetrahydrobiopterin (BH_4_) as cofactor, and molecular oxygen (O_2_) as additional substrate for catalysis^1^. Mutations in TH lead to the disease tyrosine hydroxylase deficiency, and TH is also a marker for the dopaminergic neurons that degenerate in Parkinson’s disease^2^. Thus, TH is of considerable scientific interest and a focus of intense research.

TH belongs to the BH_4_-dependent aromatic amino acid hydroxylases (AAAH), a family of enzymes that catalyze physiologically and medically important reactions. Phenylalanine hydroxylase (PAH) and tryptophan hydroxylase 1 and 2 (TPH1 and TPH2) are the rate-limiting enzymes in the degradation of excess L-phenylalanine and the production of serotonin, respectively^3^. The three enzymes are homotetramers, with each subunit consisting of a regulatory ACT domain with an unstructured N-terminal tail of different length, a catalytic domain including the iron coordinated to a 2-His-1-carboxylate facial triad motif^4^, and an oligomerization domain^3^,^5^. For PAH, structures of the full-length tetramer from rat were recently solved^6^,^7^ but for TH, only truncated forms are available. These have provided the crystal structure of the catalytic and oligomerization domains (PDB IDs 1TOH and 2XSN)^4^ and the NMR structure of the regulatory ACT domain (PDB ID 2MDA)^8^ and enabled construction of structural models for full-length TH^3^,^5^,^8^, which await experimental validation.

A complete understanding of the complex structure, catalytic mechanism, and regulatory properties of TH and other AAAHs requires protein preparations of high homogeneity. The first studies on isolated TH used native enzyme isolated from neuroendocrine tissues, purified as a partially inhibited catecholamine–enzyme complex^9^,^10^, and later strategies have provided recombinant human TH in large quantities as a pure, uninhibited enzyme with high enzymatic activity^11^,^12^. However, structural investigations of full-length TH have so far been unsuccessful. TH manifests inherent kinetic and conformational instability, which is partly counteracted by the binding of regulatory catecholamines^13^,^14^.

Recombinant proteins often aggregate either during expression and purification, or upon storage and handling at high concentrations. A common strategy to reduce aggregation is to fuse the target protein to a more stable protein, which is removed by specific proteases in the last step of the purification. In this respect, not only the yield and purity, but also properties such as solubility, conformation, and toxicity of the protein of interest may be dependent on the chosen fusion partner^15^. Nakashima *et al*. expressed TH with maltose-binding protein (MBP) as an N-terminal fusion partner already in 1991^16^. However, they discovered that Factor Xa, the protease required to separate fusion partners in the pMAL system, also cuts within the N-terminus of TH. Higgins *et al*. circumvented this problem by using a tobacco etch virus (TEV) protease-cleavable MBP-TH variant^17^. However, compared to other published data on recombinant TH^11^,^18^, they obtained an enzyme with low activity.

Since Nagatsu *et al*. first isolated TH, it has been known that the addition of Fe^2+^ stimulates TH activity^1^. Native TH has been reported to contain significant amounts of iron (0.66 mol/mol TH subunit) and zinc (0.13 mol/mol TH subunit) when isolated from bovine adrenal medulla^10^, or up to 1 mol iron/mol TH subunit when isolated from rat pheochromocytoma (PC12) cells^19^. The use of buffers containing 1 mM ethylenediaminetetra-acetic acid (EDTA) provided recombinant TH largely as an apoenzyme, with a metal content reported to be as low as 0.04 mol iron and 0.02 mol zinc per mol of TH^11^. The requirement of TH for Fe^2+^ for catalytic activity, associated to the formation of the reactive Fe(IV) = O hydroxylating intermediate^20^, appears absolute, and TH activity is inhibited by other divalent metals, such as Zn^2+^, Co^2+^ and Ni^2+ ^11. The absolute requirement for iron also applies to the important regulatory function of DA. DA, produced by DOPA decarboxylase from L-DOPA, and the other downstream catecholamines noradrenaline and adrenaline act as feedback inhibitors of TH by coordinating directly to the iron and inhibiting catalysis. Recently, the importance of the concomitant stabilizing effect of DA has also been shown to be crucial for the correct localization of TH in the brain^18^.

In the current study, we aimed to produce stable, homogeneous preparations of recombinant human TH isoform 1 (TH1) for structural investigations. Of the four human TH isoforms, originated by alternative splicing (TH1-TH4), TH1, which is very abundant in brain and aligns well with rodent TH, is the one customarily used in *in vitro* investigations^21^. We present strategies that resulted in two preparations of TH1, expressed from pET-1a vectors, with improved stability and homogeneity. These are thus more suitable for both functional and structural studies compared to TH1 expressed without a fusion partner^22^. Interestingly, the most stable preparation was a partly inhibited enzyme that contains cobalt in the active site. These purification strategies resulted in TH1 samples that provided high-quality small-angle X-ray scattering (SAXS) data and allowed the construction of structural models for the full-length enzyme.

## Results

### Cloning, expression, and purification of TH1 with different partners

**Figure 1:**
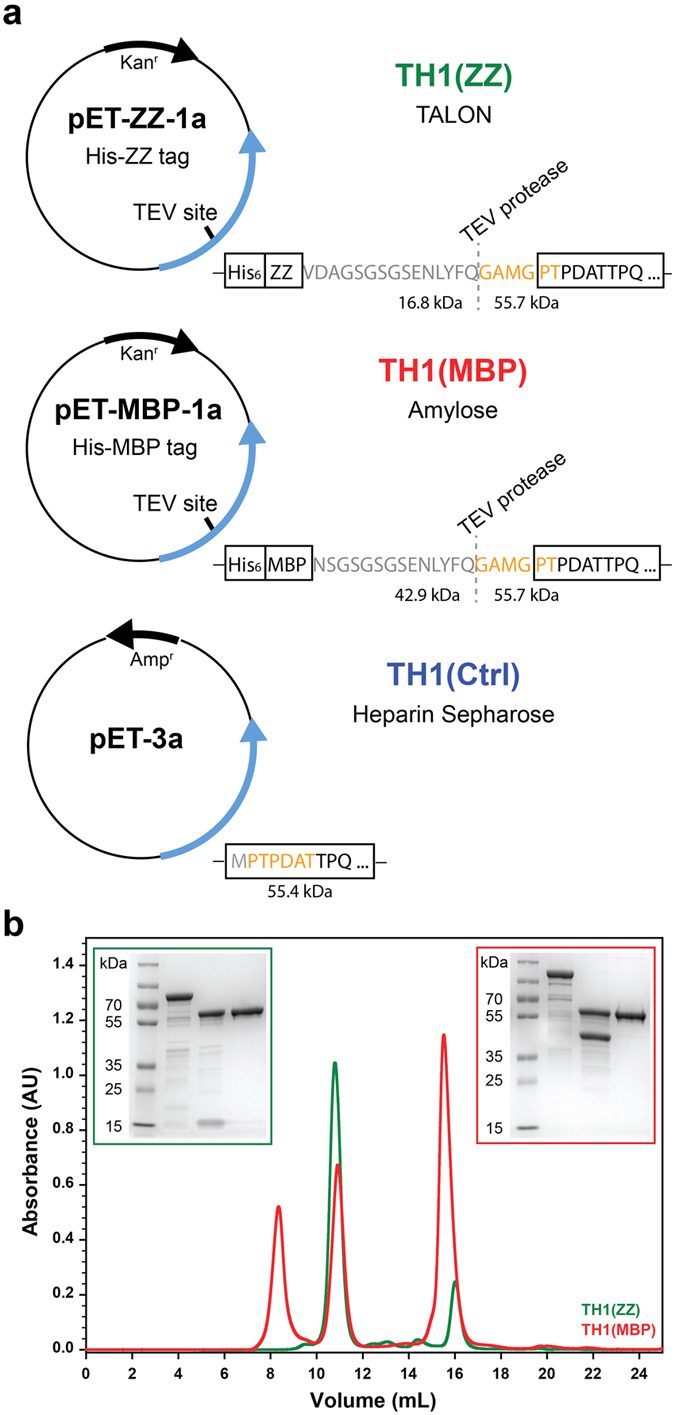
The three TH1 preparations. (**a**) Simplified illustration of vector constructs used in this study, leading to the following TH1 forms: TH1(ZZ) purified on TALON resin as His_6_-ZZ-TH1 and cleaved by TEV protease (green), TH1(MBP) purified on amylose resin as His_6_-MBP-TH1 and cleaved by TEV protease (red) and TH1(Ctrl) purified on Heparin Sepharose (blue). Open reading frames for ampicillin (Amp) or kanamycin (Kan) resistance genes and TH1 fusion proteins are shown as arrows, and cleavage sites for proteases are indicated. Amino acids of the N-termini revealed by Edman analysis are highlighted in orange. (**b**) Analytical gel filtration chromatogram of TH1(ZZ) (green) and TH1(MBP) (red) on a Superdex 200 Increase 10/300 column. The elution profile illustrates the separation of aggregates, tetrameric TH1 and the other cleavage products (fusion partner and TEV protease). Insets: SDS-PAGE of 2 μg purified protein. Lane 1: prestained protein ladder; lane 2: fusion protein; lane 3: cut fusion protein; lane 4: purified TH1.

We tested and compared recombinant human TH1 expressed without fusion partner and purified on Heparin Sepharose (TH1(Ctrl); Fig. 1a)^11^ with TH1 expressed as fusion proteins. The original TH1(Ctrl) preparations, with a flexible, unprotected N-terminal tail during expression, often show heterogeneity in the N-terminus and variable stability between different purifications. We therefore designed constructs for expressing TH1 fused *via* a TEV protease site to either His_6_-ZZ – with ZZ being a synthetic IgG-binding domain – or to His_6_-MBP. These were purified on TALON metal affinity resin *via* their His_6_-tags and, in the case of His_6_-MBP-TH1, also on amylose resin. The yield from TALON was higher for His_6_-ZZ-TH1 than for His_6_-MBP-TH1 so we preferred the former for purifications on TALON and the latter for purification on amylose resin (Fig. 1). Cleaved fusion proteins were centrifuged to remove insoluble aggregates and subjected to gel filtration to separate tetrameric TH1 from soluble aggregates and cleavage products (fusion partner and TEV protease). We observed a markedly higher proportion of soluble aggregates for TH1(MBP) than for TH1(ZZ) (Fig. 1b). Edman analysis confirmed that both TH1 proteins have an identical and complete N-terminus (Fig. 1a). Although not as good as for TH1(Ctrl), the yield of TH1(MBP) and TH1(ZZ) was still sufficiently high (4–6 mg/L culture, when using autoinduction medium).

### TH activity and time-dependent inactivation of TH1

**Figure 2:**
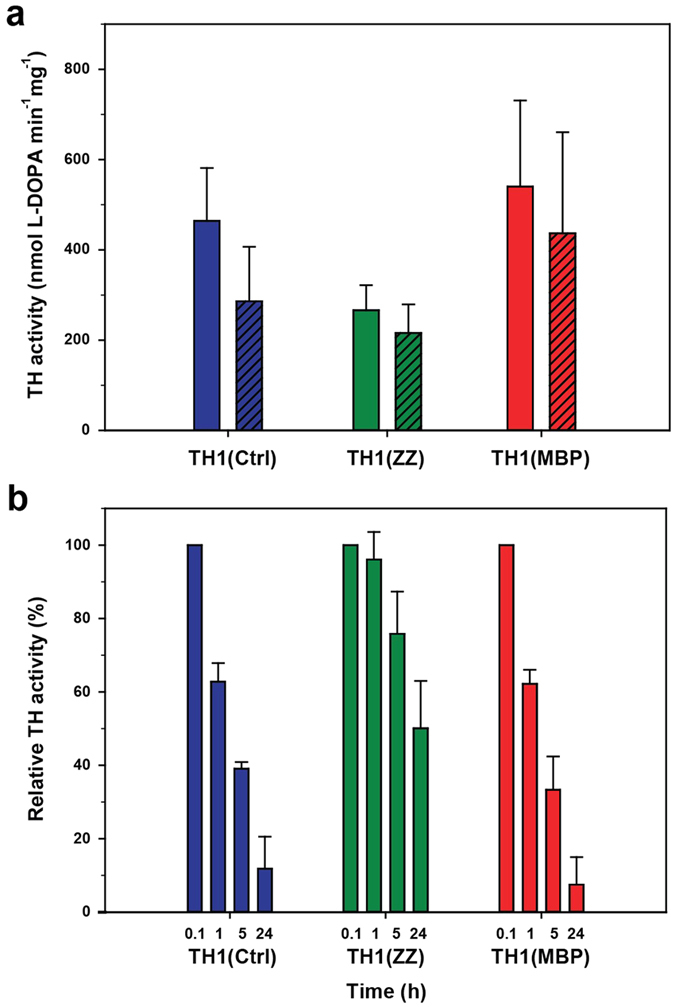
The activity of the TH1 preparations. (**a**) Specific TH1 activity of TH1(Ctrl) (blue), TH1(ZZ) (green) and TH1(MBP) (red), with (closed bars) and without (hatched bars) addition of 10 μM Fe^2+^ in the assay. The data represent the mean ± combined SD of at least four independent measurements each performed in triplicates. (**b**) Remaining TH1 activity (% of initial activity) as a function of pre-incubation time. The activity of TH1(Ctrl) (blue), TH1(ZZ) (green), and TH1(MBP) (red) was measured with 50 μM L-Tyr and 200 μM BH_4_ after 5 min, 1 h, 5 h and 24 h pre-incubation at 37 °C, pH 7.0. The data represent the mean ± combined SD of two independent measurements where each data set was performed in triplicates and normalized to the initial activity (5 min pre-incubation).

In order to determine if the activity of TH1 is affected by the used purification strategies, we measured the specific activity of the preparations using a standard reaction mixture both with and without 10 μM Fe^2+^. Addition of Fe^2+^ is customarily used in TH activity assays to ensure that maximal activity is reached^11^. As expected, the activity was higher upon addition of iron, notably for TH1(Ctrl) and TH1(MBP). Under the standard activity assay conditions, with Fe^2+^ added, TH1(MBP) showed the largest activity, followed by TH1(Ctrl) and TH1(ZZ) (Fig. 2a). However, when time-dependent loss of activity was measured upon incubation of the enzyme at 37 °C, both TH1(Ctrl) and TH1(MBP) lost >50% of their initial activity after 5 h and ≥80% after 24 h. Surprisingly, TH1(ZZ) maintained >50% of its activity up to 24 h (Fig. 2b).

### Thermal stability of TH1

**Figure 3:**
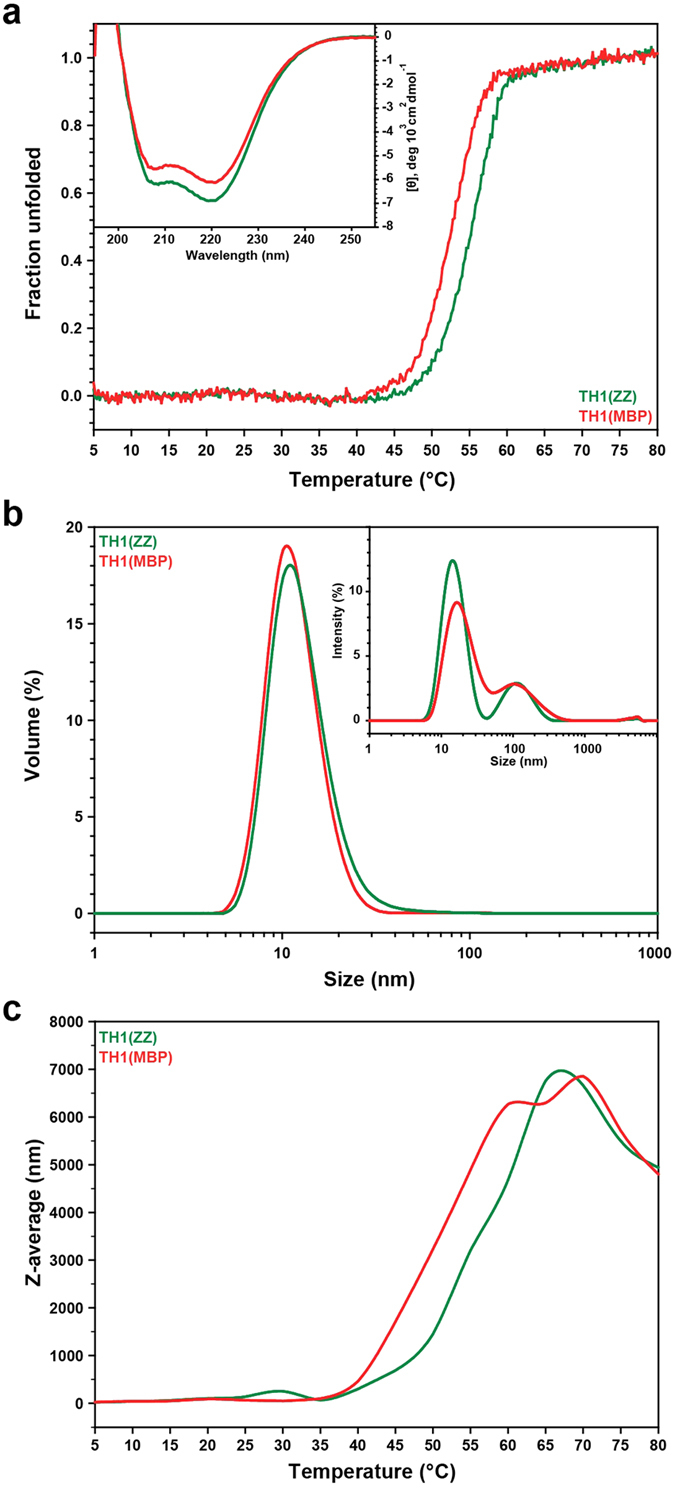
Stability of the TH1 forms. (**a**) CD-monitored (at 222 nm) thermal denaturation of TH1(ZZ) (green) and TH1(MBP) (red). TH1 (4 μM subunit) was in 10 mM Na-HEPES pH 7.0, 100 mM NaCl. The fraction unfolded TH1 data are the mean of six replicates. Inset: Far-UV CD spectra of 4 μM TH1(ZZ) (green) and TH1(MBP) (red) at 5 °C in 10 mM Na-HEPES pH 7.0, 100 mM NaCl. The data represent the mean of six replicates. *T*_m_ values and estimated α-helical content are summarized in Table 1. (**b**) DLS size distribution of TH1. Volume size distribution of TH1(ZZ) and TH1(MBP) at 5 °C in 10 mM Na-HEPES pH 7.0, 100 mM NaCl. Inset: Intensity size distribution at 5 °C. The DLS data represent the mean of three replicates. (**c**) DLS thermal stability of TH1(ZZ) and TH1(MBP). DLS thermal scans of TH1(ZZ) (green) and TH1(MBP) (red), both with 18 μM subunit in 10 mM Na-HEPES pH 7.0, 100 mM NaCl.

The conformation and thermal stability of TH1(ZZ) and TH1(MBP) were investigated by circular dichroism (CD) spectroscopy. The far-UV spectra (Fig. 3a, inset) showed local minima at 208 and 222 nm, characteristic of a well-folded helical structure. The estimated α-helical content ranged between 35% and 41% and correlated well with reported values for TH1(Ctrl) (Table 1)^23^. Thermal unfolding experiments provided the midpoint melting temperature (*T*_m_) after normalization and fitting of the profiles to a two-state model (Fig. 3a, Table 1). The results revealed that both TH1(ZZ) and TH1(MBP) were more stable than the control TH1, but surprisingly, the *T*_m_ of TH1(ZZ) was almost 3 °C higher than that of TH1(MBP) (Table 1).

### Aggregation propensity of TH1(ZZ) and TH1(MBP)

As a further characterization of the improved recombinant TH1 forms, we applied dynamic light scattering (DLS) to investigate how the hydrodynamic diameter of TH1(ZZ) and TH1(MBP) changed as a function of temperature. The intensity size distribution in DLS showed two populations at 5 °C for both TH1 preparations (Fig. 3b, inset), a large population of TH1 with a diameter of ∼15 nm, and a smaller one of ∼105 nm. The former value corresponds well to the expected diameter of TH in tetrameric structural models^3^, whereas the population with a larger diameter points to soluble oligomeric aggregates. On the other hand, the volume size distribution obtained from the DLS scans at 5 °C revealed single populations with an apparent hydrodynamic diameter of 11.9 ± 1.2 nm and 13.2 ± 1.4 nm for TH1(ZZ) and TH1(MBP), respectively (Fig. 3b). This result indicates that the highly scattering aggregates detected in the size distribution profiles are only present in tiny amounts. DLS thermal scans showed a lower aggregation propensity for TH1(ZZ) than for TH1(MBP) (Fig. 3c), supporting the results from gel filtration chromatography of cleaved fusion proteins (Fig. 1b) and the higher thermal stability for TH1(ZZ) measured by thermal scanning CD (Fig. 3a).

### Metal content in TH1 preparations

As a part of the characterization of the physicochemical properties of TH1(ZZ) and TH1(MBP), we investigated their metal content. Both TH1 forms are expressed from similar pET-1a vectors, with the only difference being the fusion partner and the type of affinity resin used during purification, *i.e.* TALON for the purification of TH1(ZZ) and amylose resin for the purification of TH1(MBP). TH1 shows an absolute requirement for iron for catalysis, while other metals have been reported to bind and inhibit TH1, leading to an inability to catalyze L-DOPA formation from L-Tyr^11^,^24^. As measured by inductively coupled plasma mass spectrometry (ICP-MS), TH1(ZZ) contained almost no iron (0.06 ± 0.07 mol/mol TH1 subunit) compared to TH1(MBP) (0.25 ± 0.18 mol/mol TH1 subunit). The specific activity of TH1(ZZ) was lowest, regardless of added Fe^2+^ (Fig. 2a), and resembles the activity of TH1(Ctrl) without added Fe^2+^, which is largely purified as an apoenzyme. We thus hypothesized that the active site might be occupied by another metal than iron, most probably cobalt, the metal that coordinates to the His tag on the TALON resin. We performed further metal quantifications and did, indeed, observe high amounts of cobalt in TH1(ZZ) (0.70 ± 0.25 mol/mol TH1 subunit), while no cobalt was detected in TH1(MBP).

### The origin of the higher stability of TH1(ZZ)

**Figure 4:**
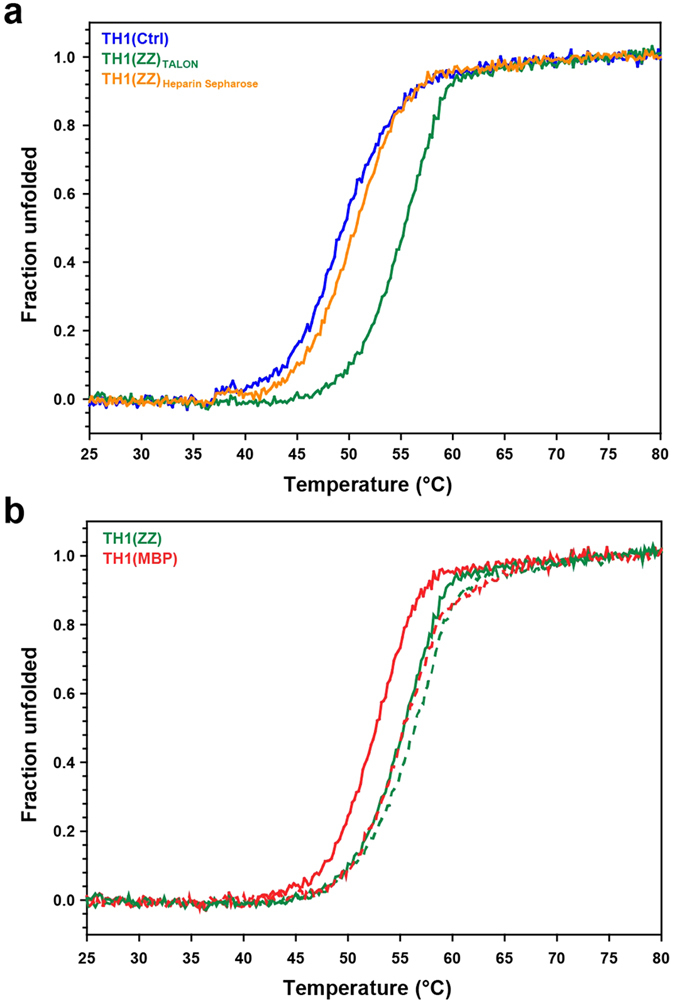
Effect of purification on metal affinity resin on TH1 stability. CD-monitored (at 222 nm) thermal denaturation of TH1 (4 μM subunit) in 10 mM Na-HEPES pH 7.0, 100 mM NaCl. (**a**) TH1 purified as a His_6_-ZZ fusion, either on TALON (TH1(ZZ)_TALON_, green) or on Heparin Sepharose (TH1(ZZ)_Heparin Sepharose_, orange). The denaturation profile for control TH1 purified without fusion partner is also shown (TH1(Ctrl), blue). The data represent the mean of six replicates. (**b**) TH1(ZZ) purified on TALON and TH1(MBP), both in the absence (solid line) and presence (dotted line) of twice the stoichiometric amounts of DA (8 μM). The data represent the mean of four replicates.

In order to investigate whether the unique high stability of TH1(ZZ) (high *T*_m_ and low aggregation propensity) is intrinsically associated to the cobalt in the active site, or to its fusion with the ZZ partner during expression and initial purification, we purified His_6_-ZZ-TH1 on Heparin Sepharose, following the purification protocol for TH1(Ctrl). This TH1, obtained after cleavage with TEV protease, had thus not been in contact with the cobalt-containing TALON resin during purification and was – as TH1(MBP) – found to be devoid of cobalt. CD-monitored thermal denaturation provided a *T*_m_-value of 50.1 ± 0.6 °C for TH1(ZZ)_Heparin Sepharose_ (Fig. 4a), 3 °C higher than the *T*_m_ for TH1(Ctrl) but ∼5 °C lower than for TH1(ZZ)_TALON_ (Table 1). This shows that the substantial increase in stability of TH1(ZZ) is conferred not by the protein construct itself, but by the purification on TALON metal affinity resin, which most likely releases cobalt to the enzyme preparation.

### Dopamine binding

An important regulatory mechanism for TH is the binding of DA to the iron in the active site that both inhibits and stabilizes TH^9^,^13^,^14^. Although TH1(ZZ) has desirable properties with regards to stability, TH1 used for functional studies must be properly regulated. Therefore, we tested whether our improved TH1 proteins can bind and be stabilized by DA using CD-monitored thermal denaturation. The *T*_m_ for TH1(MBP), as also recently shown by Korner *et al*. using differential scanning fluorimetry^18^, increased by approximately 3 °C upon the addition of 8 μM DA, *i.e.* a saturating concentration based on the IC_50_ of 1.9 μM for DA^25^. The *T*_m_ of DA-bound TH1(MBP) is similar to that of purified Co-bound TH1(ZZ), which was only slightly stabilized by DA (≤1 °C) under similar conditions (Fig. 4b).

### The SAXS solution structure of full-length TH1

**Figure 5:**
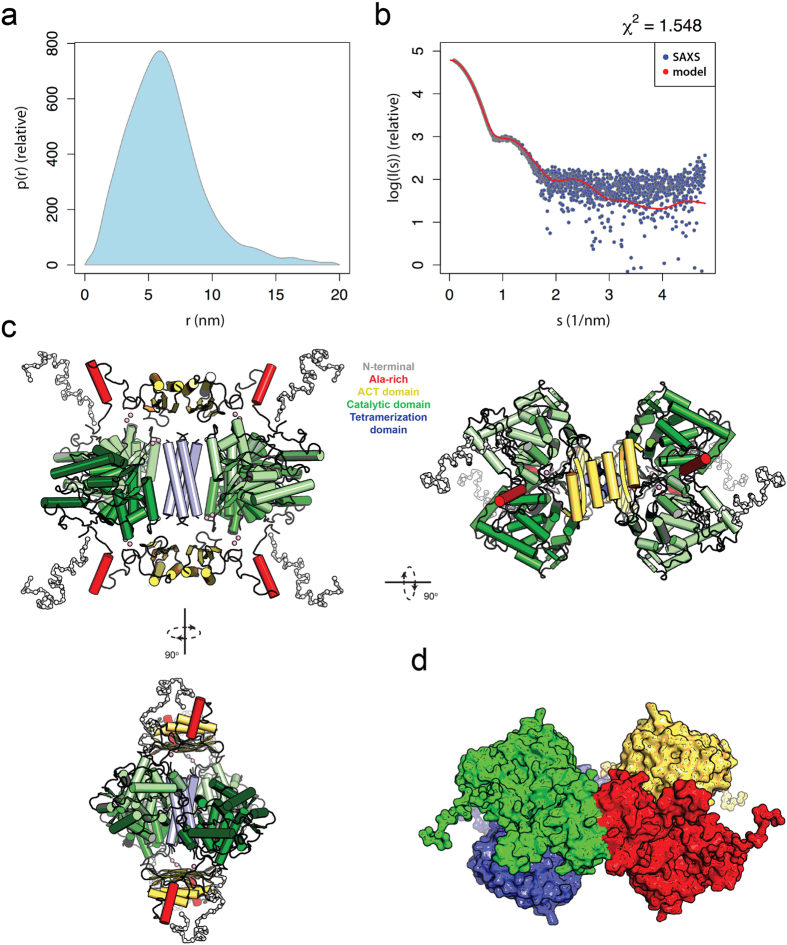
Small-angle X-ray scattering of TH1(ZZ). (**a**) Distance distribution function. (**b**) Raw X-ray scattering data (blue dots) and the fit of the model (red line). (**c**) Model of TH obtained by hybrid modelling shown in three orientations. Coloring depicts the various structural elements and domains of TH1: N-terminal tails (Met1-Asp44); Ala-rich region of N-terminal region (Ala45-Ala70); ACT domain (Val71-Arg156); catalytic domains (Ser157-Asp470); and tetramerization helices (Ser471-Gly497); numbering corresponds to human TH1. (**d**) Model in surface representation with colors representing the four subunits in the complex.

Based on the good conformational properties of TH1(MBP) and TH1(ZZ), we expected that these enzyme forms would be amenable to structural characterization and proceeded to perform synchrotron SAXS measurements. Due to its higher stability, we argued that TH1(ZZ) was the most promising candidate for further structural studies. Indeed, several preparations of this sample were tested that presented little aggregation or radiation damage. While both TH1(MBP) and TH1(ZZ) provided similar scattering curves, notably TH1(MBP) presented some aggregation (see Supplementary Fig. S1). SAXS data collection and parameters obtained for TH1(ZZ) are shown in Table 2. These data were used for modelling of the full-length solution structure of TH1. The MW based on forward scattering confirmed the presence of a tetramer, with an experimental radius of gyration *R*_g_ = 4.74 ± 0.33 nm and a maximum interatomic distance *D*_*max*_ = 20 nm, indicating an elongated shape. This conformation is supported by both the distance distribution function (Fig. 5a) and *ab initio* modelling. The chain-like *ab initio* shape, built using the expected P222 symmetry, shows approximate dimensions of 17 × 9 × 9 nm, with a dense equatorial core, corresponding to the catalytic and oligomerization domains, and two smaller densities on each side of the core, extending out into the solvent (see Supplementary Fig. S2).

To obtain a more detailed structure of the complex we employed a multiscale modelling protocol using (1) components determined previously from crystallography and NMR, (2) homology modelling, and (3) protein structure folding through molecular simulations. These components were combined through SAXS-based simulated annealing protocols (see Methods). The 70-amino-acid N-terminal sequence was divided into three segments: (1) residues 1–35, (2) residues 36–44, and (3) residues 45–70. Residues 1–35 were modelled *ab initio* during the simulated annealing protocol of the full complex using BUNCH^26^. Residues 36–44 contain a motif that aligns well with the corresponding residues in PAH (see Supplementary Fig. S3). Since these residues are resolved in the PAH crystal structure^27^, we used homology modelling to obtain coordinates for this segment in TH. Residues 45–70 contain an Ala-rich segment, which we suspected could obtain a folded structure due to the ability of poly-Ala peptides to form α-helices. This Ala-rich N-terminal segment (Ala45-Ala70) was issued to extensive replica exchange molecular dynamics (REMD) simulations to explore a likely three-dimensional structure (see Methods). Cluster analysis based on pairwise structural deviations grouped the 10,000 conformations into five distinct clusters. 9,572 of the 10,000 conformations were grouped into one major cluster (cluster 1) dominated by members with high α-helix propensity and a compact structure with a mean *R*_*g*_ of 0.88 nm (SD = 0.06 nm). A majority of the cluster members showed an α-helix at residues Lys47-Ala56 (see Supplementary Fig. S4). In contrast, the remaining 428 conformations (clusters 2–5) showed a more elongated configuration with a mean *R*_*g*_ of 1.44 nm (SD = 0.21 nm), also showing several members with an α-helix in the same region as for cluster 1. The cluster representative from cluster 1 contains an α-helix from Lys47 to Ala58 and a random coil from Val60 to Ala70. This configuration is in agreement with secondary structure predictions using PSIPRED^28^, which suggest Arg46-Ala59 to be an α-helix. This fragment of the N-terminal segment was connected to the ACT domains for SAXS-based rigid body modelling.

SAXS-based rigid body modelling was performed with BUNCH using P222 symmetry with the subunit chain built up from residues 1–35 (unstructured); residues 36–44 covering over the active site of the catalytic domains modelled through homology with equivalent residues from PAH^6^; residues 45–70 folded through REMD simulations described above; ACT domains obtained from the NMR structure^8^; the catalytic and oligomerization domains derived from crystallography^4^. The ACT, catalytic domains, and tetramerization domains were connected with flexible linkers allowing these domains to move relative to each other during simulated annealing. The simulations yielded a model with an overall very good fit to the raw SAXS data with χ^2^ of 1.55 (Fig. 5b). The model shows a tetramer, in which the catalytic domains orient in a similar way as in the crystal structure (PDB ID 2XSN), but with a tilt between the two dimers, making an out-of-plane tetramer (see Supplementary Fig. S5 for comparison with in-plane configuration in the crystal structure). The ACT domains are oriented above the tetramerization helix bundle, with the N-terminus of each ACT domain pointing towards the corresponding catalytic domain active site.

**Figure 6:**
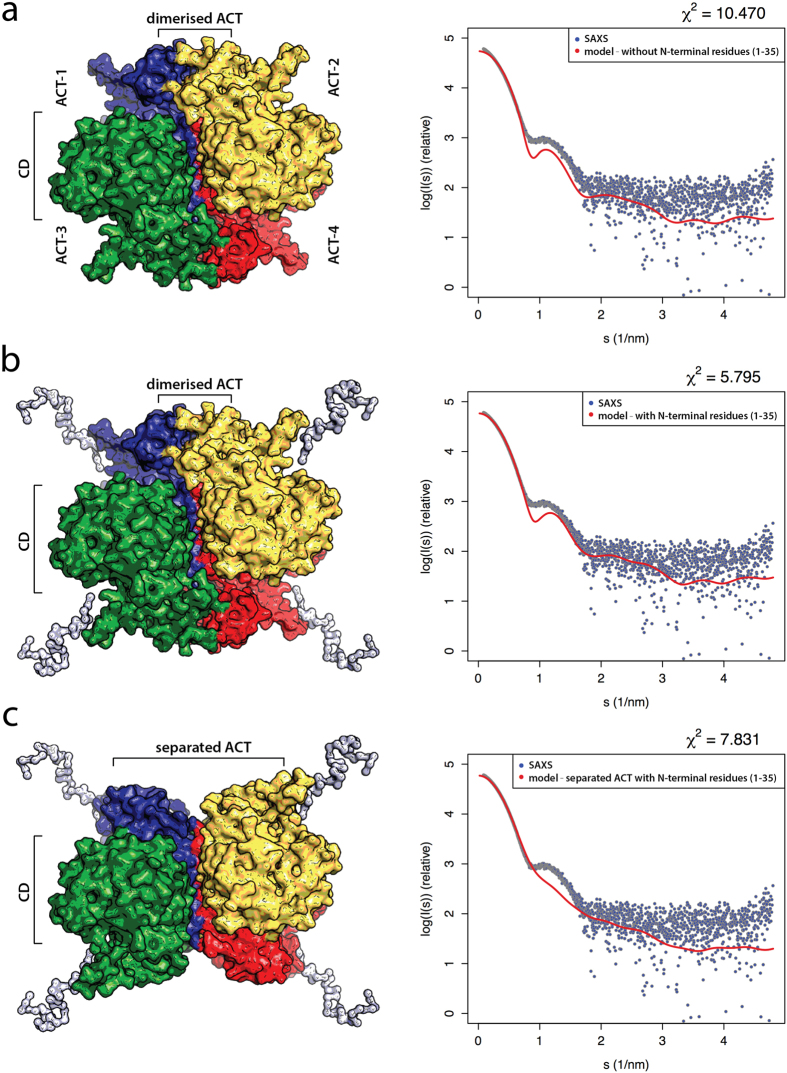
Evaluation of models. The models were evaluated using the tetrameric organization of catalytic domains (CD) as seen in the crystal structures (shown for human TH, PDB ID 2XSN) combined with dimerized ACT domains without (**a**) and with (**b**) N-terminal residues (1–35). These models without adjusting the positions of the CD provide χ^2^ values of 10.5 and 5.8, respectively. The model with ACT domains separated (**c**) shows a clear difference in the SAXS profile.

The importance of the disordered N-terminal tails of the model on the fit to the SAXS data is apparent; removing residues Met1-Ile35 provides a worse fit to the SAXS curves with a χ^2^ of 2.0. This supports the notion that the flexible N-terminal tails extend into bulk solvent from the protein core. Furthermore, modelling of TH based on the tetrameric organization of the catalytic and tetramerization domains (as seen in the crystal structures, PDB ID 2XSN and 1TOH) combined with the NMR structures of the dimerized ACT domains (PDB ID 2MDA) without rigid body movements of the catalytic domains and without the N-terminal residues (1–35) gives a suboptimal fit with a χ^2^ of 10.5 (Fig. 6a). Including the N-terminal residues improves the agreement with the SAXS data (χ^2^ of 5.8; Fig. 6b), but the fit is much worse than for the model with the catalytic domains adjusted (out-of-plane as shown in Fig. 5). Fitting was also inadequate when the dimerized ACT domains were substituted by separated ACT domains, as encountered in the recently solved crystal structure of inactivated PAH (PDB ID 5DEN)^6^ (Fig. 6c). Also in this case a large increase in χ^2^ is observed (to 7.83), supporting our SAXS-derived model.

SAXS measurements were also carried out with TH1(MBP) in order to analyze the structural effects of DA binding. The results in the absence and presence of DA indicate that DA binding does not induce large conformational changes in TH1 (see Supplementary Fig. S1), in agreement with H/D exchange studies showing that DA binding mainly affects the N-terminus and some residues close to the active site^29^.

## Discussion

### Improved TH1 purifications

The preparations of TH1 characterized in this work show differences in TH1 activity and thermal stability depending on the choice of fusion partner and/or type of affinity chromatography resin. Compared to the control TH1, which has no fusion partner and is purified on Heparin Sepharose, our new preparations have higher thermal stability and are less prone to aggregation, most likely due to a more homogeneous N-terminal region. The N-terminal tail of TH1 is very flexible, and residues 1-43 were shown to have a disordered conformation by solution NMR^8^, CD, and molecular dynamics simulations^30^, rendering it vulnerable to proteolytic degradation. To overcome this problem, we designed several fusion proteins of TH1, all containing the fusion partner at the N-terminal end of TH1. In addition to facilitating purification, the fusion partner MBP can enhance solubility and promote folding by acting as a general molecular chaperone. The latter is thought to be due to MBP binding the protein of interest *via* its exposed hydrophobic residues and by MBP recruiting chaperones, like GroEL, present in the *E. coli* host cell^31^. Differently to the case with fusion proteins of MBP with a Factor Xa cleavage site^16^, we obtained full-length TH1(MBP) preparations with an intact N-terminus and a high activity from the His_6_-MBP-TH1 fusion protein cleaved by TEV protease. This protease is also used with TH1 from pET-ZZ-1a that produces TH1 with His_6_-ZZ, a smaller fusion tag of 17 kDa (Fig. 1a). The ZZ fusion may pose less of a metabolic burden on the host cell than the larger fusion partner MBP, and the ZZ domain has also been used to solubilize proteins that tend to aggregate during expression^32^, probably explaining the higher yield of TH1 obtained from His_6_-ZZ-TH1 than from His_6_-MBP-TH1 purified on TALON.

### Cobalt-bound TH1(ZZ) is remarkably stable

Although it is generally not recommended to purify metal-containing proteins on metal affinity resins, we have previously successfully purified several bacterial forms of PAH as His_6_-tagged fusion proteins on TALON columns^33^,^34^. One of these was, indeed, purified almost solely as an apoenzyme, but it displayed very high activity upon reconstitution with iron^33^. This was, however, not the case for TH1. The increase in enzymatic activity of TH1(ZZ) purified on TALON upon addition of iron in the assay was not significant and much lower than that observed for both TH1(Ctrl) and TH1(MBP) (Fig. 2a). However, the activity and conformation of TH1(ZZ) show increased thermostability. As measured by ICP-MS, TH1(ZZ) contains cobalt in substantial amounts, and we assumed that the cobalt – originating from the purification resin – is the inhibiting and stabilizing element^24^. The enzyme purified from the same His_6_-ZZ-TH1 construct, but on Heparin Sepharose instead of TALON, did not exhibit this increase in stability (Fig. 4a). Indeed, it has also been shown for other metalloenzymes that their cobalt-bound state is more stable than the iron-bound form^35^,^36^. Although we acknowledge the fact that a cobalt-containing TH1(ZZ) is not suited for specific functional and regulatory studies, its improved stability makes it a promising candidate protein for structural studies of full-length TH1 — a long awaited milestone in the study of this enzyme. For functional studies, we may prefer to use TH1(MBP), an enzyme that presents high specific activity, a homogeneous N-terminus, and stabilization by the natural end-product inhibitor DA.

### The SAXS-derived structure of full-length TH1

We obtained the first experimental three-dimensional structure of full-length tetrameric TH1 by SAXS using TH1(ZZ) preparations. The SAXS structure resembles that of structural models of TH1 prepared recently based on combinations of crystal and NMR structures of the different domains^3^,^8^. Nevertheless, whereas the crystal structure of tetrameric catalytic and oligomerization domains presents all four catalytic domains in-plane^4^, the SAXS data fit best to a model in which the catalytic domains are out-of-plane (see Supplementary Fig. S5). Structural differences between solution and crystalline states have been reported for several proteins^6^,^7^,^37^. For instance, the recent solution structure of PAH^7^ revealed a V-shaped asymmetry in the tetramer that adds to the asymmetry in the dispositions of the domains that is already encountered in the crystal structures of tetrameric PAH^6^,^38^ and that has been interpreted to be related to the flexible nature of an allosteric tetramer that shows positive cooperativity for its substrate^7^. Despite devoid of the positive cooperativity for its substrate, characteristic of PAH^3^,^5^, allosteric effects of the natural BH_4_ cofactor on TH activity have been described, including both positive cooperativity that is counteracted by DA neurotoxin^39^, as well as negative cooperativity^40^, which might also been related to allosteric and/or regulatory conformational changes in TH.

Even though TH and PAH have a very similar geometry and size with respect to the core ACT, catalytic and regulatory domains, the dimensions of TH1 and PAH are rather different. TH1 adopts a more elongated shape (*R*_g_ = 4.74 ± 0.33 nm, *D*_*max*_ = 20 nm) than full-length PAH (*R*_g_ = 4.05 nm, *D*_*max*_ = 11.7 nm)^6^, which is only 10% smaller in MW than TH1, revealing the large impact of the unstructured N-terminal region on the shape of TH1 (Fig. 5). Our SAXS-based structure contributes to the structural and functional understanding of TH and complements previous models by including the modelled N-terminal region (residues 1–70; see Supplementary Fig. S3). Previous conformational studies using the isolated 1-43 residues from the N-terminal tail have shown that it presents intrinsic disorder, but that it has a tendency to adopt α-helical secondary structure, *e.g.* upon interaction with membranes^30^. This mobile and unstructured tail is the part of TH1 that contains the phosphorylation sites Thr8, Ser19, Ser31, and Ser40 that regulate activity and interactions with partners^21^,^41^. Interestingly, the modelled structure maintains Ser40 close to the entrance of the active site, at a suitable distance to interact with DA and other catecholamines, and to contribute to the high-affinity binding of these feedback inhibitors, an interaction that is released upon Ser40 phosphorylation by cAMP dependent protein kinase^42^. Furthermore, the SAXS model reveals an arrangement of the N-terminal tail pointing out from the globular domains, which is in agreement with the site of binding of the 14-3-3γ protein in the phosphoSer19-TH:14-3-3γ complex, as observed by EM^43^. Actually, the Ala-rich motif also appears as a well-located docking interface for partner protein interactions that may modulate the localization, function and stability of TH. Recently, affinity capture-MS data on the human interactome has revealed a large number of protein-protein interactions of TH, as expected for a tightly regulated enzyme^44^ and the present structural model of the N-terminal region of TH certainly shows features compatible with a multi-partner protein. Finally, the SAXS data and derived model validate a dimeric disposition of the ACT domains (Fig. 6c). A recent study has also shown that the ACT domains of TPH1 form a structural homodimer, indicating that a dimeric arrangement of adjacent domains may also be a feature in the full-length TPH^45^. This is similar to the arrangement found for PAH only when it is activated by its substrate L-Phe. In the inactivated PAH the ACT domains do not interact with one another and transition to dimeric state is associated to activation by L-Phe^6^,^7^. Neither TH nor the TPHs are activated by their substrates and thus such a monomer-dimer exchange may not take place in these hydroxylases. Importantly, a dimerized configuration of the regulatory domains seems to provide increased stability^45^.

In conclusion, we present the preparation of highly pure, stable forms of full-length TH1. TH1(MBP) shows a high activity level, is activated by Fe^2+^, and is stabilized by regulatory DA, but it is less stable than TH1(ZZ). On the other hand, TH1(ZZ) is stabilized by cobalt, which largely inhibits its activity but protects the enzyme from denaturation and aggregation. This preparation allowed the characterization of the structural organization of full-length TH by SAXS, which has until now been hindered, most likely by the low stability and large heterogeneity in earlier TH preparations. The SAXS-derived structure presents full-length TH with dimeric ACT domains and an elongated conformation due to a large influence of the unstructured N-terminal region compared with PAH. This long N-terminal region (residues 1–70) is important for regulation of TH by phosphorylation and for interaction with partners.

## Methods

### Cloning strategies

The pET-TH expression vector, generated by Le Bourdellès *et al*.^22^ by cloning the *TH1* cDNA into pET-3a, was used as a basis for constructing other expression vectors. Insertion of *TH1* into both pET-ZZ-1a and pET-MBP-1a was performed by PCR using the primers 5′- GCTTCCATGGGACCCACCCCCGA-3′ and 5′-GCTTGGTACCCAGTGCAGGACCA-3′ and the restriction enzymes NcoI and Acc65I, resulting in an extra glycine residue in position 2 and the residues glycine and alanine in front of the starting methionine after removal of the fusion partner. Correct sequences were verified by sequencing.

### Expression and purification of recombinant TH1 proteins

TH1 protein variants were overexpressed in *E. coli* BL21-CodonPlus(DE3)RIL. Briefly, cells were grown at 28 °C and 200 rpm in LB medium with 1 mM isopropyl β-D-thiogalactoside added for induction at an OD_600nm_ of 0.6 for 6 h, or in autoinduction medium for 24 h, supplemented with 0.2–1 mM Fe^2+^ (as ferrous ammonium sulfate) and the appropriate antibiotic (Fig. 1a). Fusion proteins were purified at 4 °C using the appropriate affinity chromatography resin (Fig. 1a). The equilibration and wash buffers used were 20 mM Na-HEPES pH 7.0, 200 mM NaCl for amylose resin, 20 mM Na-phosphate pH 7.0, 300 mM NaCl, 15 mM imidazole for TALON^®^ Superflow™ Metal Affinity Resin, and 20 mM Tris-HCl pH 7.5, 1 mM EDTA, 1 mM dithiothreitol (DTT), 5% sucrose (w/v) with Heparin Sepharose. Bacterial cells were disrupted by sonication (3 × 45 s pulses at 20% power) or French press in buffers with 1 mM phenylmethanesulfonyl fluoride, 10 mM benzamidine and cOmplete™ EDTA-free Protease Inhibitor Cocktail. The clarified extract was applied to the resin at 1–2 mL/min. After washing, bound proteins were eluted using wash buffers with either 15 mM maltose (amylose resin), 135–250 mM imidazole (TALON Superflow resin), or 0.5 M NaCl (Heparin Sepharose). Imidazole was removed from eluted fractions by buffer exchange.

The fusion protein, incubated with TEV protease^46^ at 4 °C for 1–3 h, was loaded into a HiLoad^TM^ Superdex^TM^ 200 prep grade column (1.6 cm × 60 cm) to purify soluble tetrameric TH1 or into a Superdex 200 Increase GL column (1.0 cm × 30 cm) for analytical purposes. Protein concentration was measured at 280 nm, using the theoretical molar extinction coefficient of 40,715 M^−1^ cm^−1^.

### N-terminal sequencing

N-terminal sequencing was performed by the Proteome Factory AG (Berlin, Germany), using Edman analysis. Samples were prepared according to standard protocols for semidry blotting on a polyvinylidene difluoride membrane. TH1(Ctrl), TH1(ZZ), and TH1(MBP) samples (Fig. 1a) were sequenced with 5 steps to determine a 5-amino-acid sequence.

### TH activity assay

TH activity was measured as described^11^ with minor modifications. Briefly, TH1 (0.1 mg/mL, 1.8 μM subunit) was pre-incubated with 1% BSA (w/v) in 20 mM Na-HEPES pH 7.0, 200 mM NaCl on ice. 5 μL aliquots were incubated for 1 min in a standard reaction mixture containing 50 μM L-Tyr, 0.1 mg/mL catalase, and 10 μM Fe^2+^ in 40 mM Na-HEPES pH 7.0, or in this mix without Fe^2+^. The reaction was started by adding 200 μM BH_4_ in 2 mM DTT, stopped after 5 min with 2% acetic acid in ethanol (v/v), frozen at -20 °C for at least 30 min, and centrifuged for 15 min at 4 °C and 18,000 × g. The amount of L-DOPA was measured in the supernatants by high-performance liquid chromatography analysis with fluorescence detection, as described^47^. For the time-dependent TH activity assay, the pre-incubation was at 37 °C and included 1 μM Fe^2+^, and aliquots were taken out after 5 min, 1 h, 5 h, and 24 h and measured in the standard reaction mix.

### Circular dichroism spectroscopy

CD measurements were performed on a Jasco J-810 spectropolarimeter equipped with a CDF-426S Peltier element for temperature control. Far-UV CD spectra between 180–260 nm were acquired at 5 °C by using 0.2 nm data pitch, 2 nm band width, 20 nm/min scan speed, and accumulation of 4 spectra. CD spectra of TH1 (4 μM subunit) in a 1 mm quartz cell were acquired in 10 mM Na-HEPES pH 7.0, 100 mM NaCl. Baseline buffer spectra were subtracted. Thermal denaturation of TH1 (4 μM subunit) was recorded from 5 to 80 °C at 222 nm with a scan rate of 2 °C/min and 0.2 °C data pitch. DA binding to TH1(ZZ) and TH1(MBP) was tested by adding twice the stoichiometric amount of DA. All thermal scans were normalized, fitted to a two-state unfolding model^48^, and further converted to fraction of unfolded protein as described^49^. CDNN^50^ was used to estimate secondary structure content.

### Dynamic light scattering

DLS was performed on a Malvern Zetasizer Nano ZS instrument, using a HeNe laser at 633 nm and a fixed scattering angle of 173° (back scatter). Temperature scans were recorded from 5 to 80 °C, with a temperature interval of 5 °C. TH1 preparations were diluted to 1 mg/mL (18 μM subunit) in 10 mM Na-HEPES pH 7.0, 100 mM NaCl. Data analysis was performed on intensity and volume size distribution curves and the Z-average size using the Malvern DTS software.

### Metal measurements

Iron and cobalt content of the TH1 preparations was measured by ICP-MS with a microwave digestion method. Briefly, TH1 (3 mg/mL, 54 μM subunit) was mixed with ultrapure nitric acid (HNO_3_, 60%) and hydrogen peroxide (H_2_O_2_, 30%) in a 5:5:2 v/v/v ratio. Using the Milestone 1200 MEGA (Sorisole, Italy), complete TH1 digestion was achieved by a six-stage program and a maximum microwave power of 600 W. 20 mM Na-HEPES pH 7.0, 200 mM NaCl and Seronorm^TM^ Trace Elements Serum L-1 (SERO) prepared in the same way were used as blank and quality control of the digestion method, respectively. The digested samples (n = 3 for each TH1 form) were transferred to metal-free tubes and quantitatively further diluted by a factor of 32.6 with Milli-Q water. Sample introduction into the ICP-MS was performed by the SC-FAST (Elemental Scientific) fully automated sample introduction system, in combination with an online internal standard addition of 1 μg/L indium solution to monitor and correct for instrumental fluctuations. Calibration standard solutions were prepared from certified single-element standard solutions (Spectrapure Standards AS). Analysis was done with the Thermo Finnigan Element 2 high-resolution magnetic sector field ICP-MS. The ICP-MS measuring accuracy and calibration curves were monitored by the standard reference material SPS-SW2 (Spectrapure Standards AS).

### SAXS measurements

SAXS measurements were carried out on the EMBL P12 synchrotron BioSAXS beamline^51^ at PETRA III/DESY, Hamburg, Germany on TH1(ZZ) and TH1(MBP) in 20 mM Na-HEPES pH 7.0, 200 mM NaCl (0.5–2.0 mg/mL, 9–36 μM subunit). DA in stoichiometric amounts compared to an enzyme subunit was added, when indicated. Data were collected at 20 °C using an X-ray wavelength of 0.124 nm and an exposure time of 45 ms/frame. 20 consecutive frames were collected for each sample; the sample was flowing through the capillary during the measurement. Frames were controlled for radiation damage and averaged. The data were recorded using a PILATUS 2M detector (Dectris, Baden, Switzerland) at a sample-detector distance of 3.0 m, covering a momentum transfer range, s (4πsinθ/λ) of 0.02–4.8 nm^−1^, where 2θ is the scattering angle and λ the wavelength and s is in units of nm^−1^.

Data processing and analysis were carried out using the ATSAS package^52^. Solvent scattering was identically measured from the corresponding buffer before and after each sample, and the average background scattering was subtracted with PRIMUS^53^. *R*_*g*_ was determined using Guinier analysis. The maximum particle dimension *D*_*max*_ and the distance distribution function *p*(*r*) were calculated using GNOM^54^. Molecular weights were estimated based on forward scattering I(0) from the sample, compared to standard samples of either glucose isomerase or BSA.

### Structural modelling

To obtain unbiased shape information for full-length TH1, chain-like models were built *ab initio* using GASBOR^55^. P222 symmetry, as seen in crystal structures of tetrameric species of truncated TH and homologues, was used during modelling.

BUNCH was used for SAXS-based rigid body modelling of TH1, using existing crystal and NMR structures as rigid body subunits interconnected by flexible loops. We divided the TH1 subunit chain into 3 rigid bodies interconnected by flexible loops: (1) the tetramerization helix (coordinates from PDB ID 1TOH^4^); (2) catalytic domain (coordinates from PDB ID 1TOH); (3) ACT domain (atomic coordinates obtained from PDB ID 2MDA^8^). To maintain known interfaces (from the crystal and NMR structures) between domains in the simulated annealing protocol we employed distance restraints between (1) two adjacent ACT domains, (2) two adjacent catalytic domains, and (3) the tetramerization bundle. CRYSOL^56^ was subsequently used to calculate the final fit to the experimental SAXS data. P222 symmetry and default values for the simulated annealing protocols in BUNCH were used, with 100 temperature steps and a maximum of 24,850 iterations at each temperature.

The Ala-rich N-terminal segment Ala45-Ala70 upstream from the ACT domain in TH was issued to extensive replica exchange molecular dynamics simulations^57^ to obtain a representative three-dimensional structure of this fragment. A linear all-atom model of the fragment was generated using AmberTools, employing the Amber99SB force field^58^ and implicit solvent model using generalized born^59^. The replica exchange simulations were run over 20 temperatures spanning from 270 to 509 K. Each replica was heated to its respective starting temperature during 200 ps. The replica exchange simulations consisted of 10,000 exchanges, each of 4 ps (2 × 10^7^ steps for each replica). The simulations were carried out using multisander in the Amber package. The resulting conformations at 300 K were extracted and clustered using cpptraj^60^. The representative structures from the most frequently populated cluster were extracted and included adjacent to the ACT domains in the model prior to the SAXS-based rigid body simulation. Independent of the replica exchange simulation, PSIPRED was used to predict the secondary structure elements in this fragment. The N-terminal residues Gly36-Asp44 of TH1 align well with the equivalent residues of PAH (see Supplementary Fig. S3), with high sequence identity, including the motif [SXIED]. Based on this alignment, the residues Gly36-Ala45 were modelled in TH1 corresponding to the PAH structure (PDB ID 5DEN)^6^.

## Additional Information

**How to cite this article**: Bezem, M. T. *et al*. Stable preparations of tyrosine hydroxylase provide the solution structure of the full-length enzyme. *Sci. Rep.* **6**, 30390; doi: 10.1038/srep30390 (2016).

## Supplementary Material

Supplementary Information

## Figures and Tables

**Table 1:** Alpha-helical content and thermal stability of the different TH1 constructs.

	Circular dichroism	
	α-helix content (%)	*T*_m_(°C)
TH1(Ctrl)	42 ± 2^23^	47^23^
TH1(ZZ)_TALON_	39.8 ± 9.1	55.1 ± 0.6
TH1(MBP)	34.9 ± 4.8	52.5 ± 0.3
TH1(ZZ)_Heparin Sepharose_	40.7 ± 13.8	50.5 ± 0.3

In all cases, n = 6.

**Table 2:** SAXS data-collection and scattering-derived parameters.

SAXS analyses	
Data-collection parameters	
Instrument	P12 beam line (PETRA-III, DESY)
Wavelength (nm)	0.124
*s* range (nm^−1^)	0.02–4.8
Exposure time (s)	0.045
Concentration range (mg/mL)	0.5–2.0
Temperature (°C)	20
Structural parameters	
I(0) (relative) [from *p*(r)]*	63,360.0
*R_g_* (nm) [from *p*(r)]	4.93
I(0) (relative) [from Guinier]*	62,494.4 ± 124.65
*R_g_* (nm) [from Guinier]	4.74 ± 0.33
*D_max_* (nm)	20.0
Porod volume estimate (nm^3^)	519.79
Dry volume of a monomer calculated from sequence (nm^3^)	67.56
Molecular mass determination	
Molecular mass *M*_r_ (kDa) [from I(0) using *p*(r)]	287.8
Molecular mass *M*_r_ (kDa) [from I(0) using Guinier]	283.8
Calculated monomeric *M*_r_ from sequence	55.8
Software employed	
Primary data reduction	PRIMUS
Data processing	PRIMUS
*Ab initio* analysis	GASBOR
Validation and averaging	PRIMUS
Rigid-body modelling	BUNCH
Computation of model intensities	CRYSOL
Three-dimensional graphics representation	PyMOL

^*^After normalization to a concentration of 1 mg/mL.
