# Supplementary material for: Stable preparations of tyrosine hydroxylase provide the solution structure of the full-length enzyme
Source: Sci Rep. 2016 Jul 27;6:30390. doi: 10.1038/srep30390 (PMC4961952; doi:10.1038/srep30390)
Supplement: Supplementary Information [file srep30390-s1.pdf]

Supplementary Information

Stable preparations of tyrosine hydroxylase provide the solution structure of the full-length enzyme

Maria T. Bezem+, Anne Baumann+, Lars Skjærven, Romain Meyer, Petri Kursula, Aurora Martinez, Marte I. Flydal

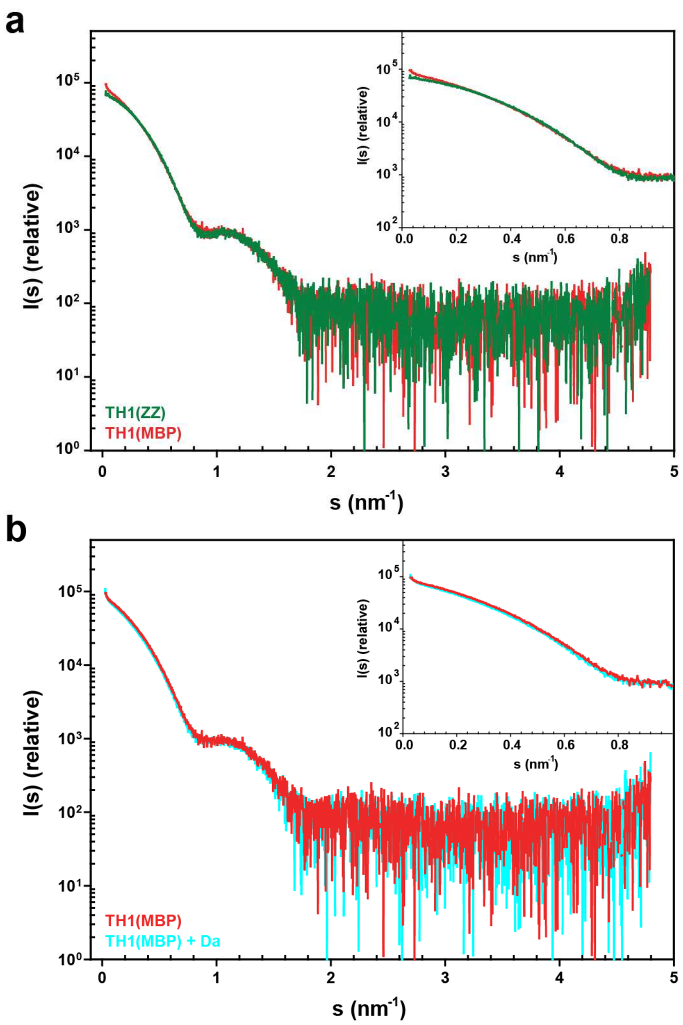

Supplementary Figure S1: Small-angle X-ray scattering. (a) SAXS curves of TH1(ZZ) (green) and TH1(MBP) (red). (b) SAXS curves of TH1(MBP) (red) and TH1(MBP) in the presence (cyan) of stoichiometric amounts of DA.

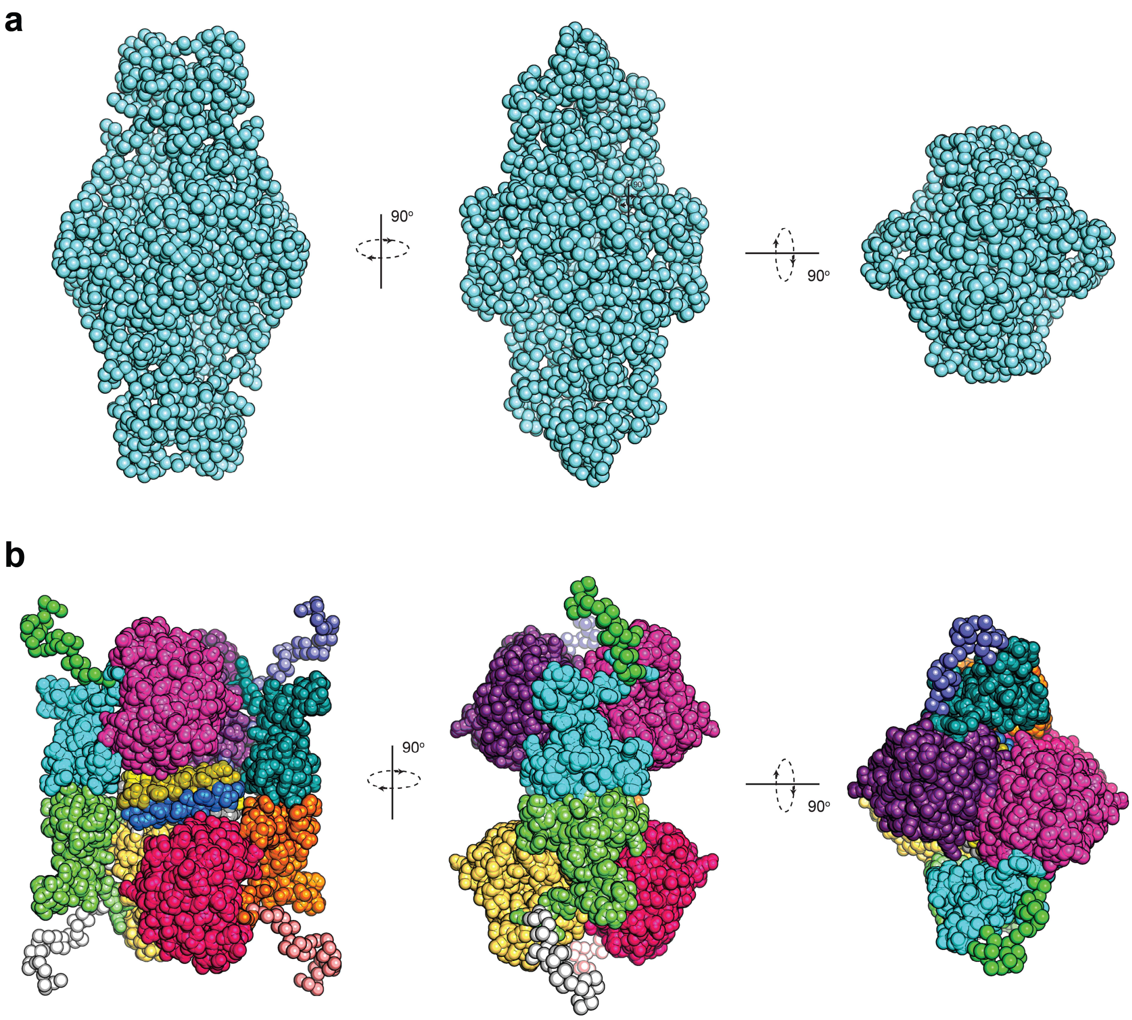

Supplementary Figure S2: Comparison of (a) *ab initio* model, and (b) hybrid model of TH1(ZZ). Dimensions of the *ab initio* model are ~17x9x9 nm and the model yields a fit to the experimental scattering data with a χ^2 value of 0.74. Dimensions of hybrid model are 15x12x10 (with a χ^2 of 1.55). Coloring represent the various structural elements and domains of TH1 used in the rigid body modeling protocol.

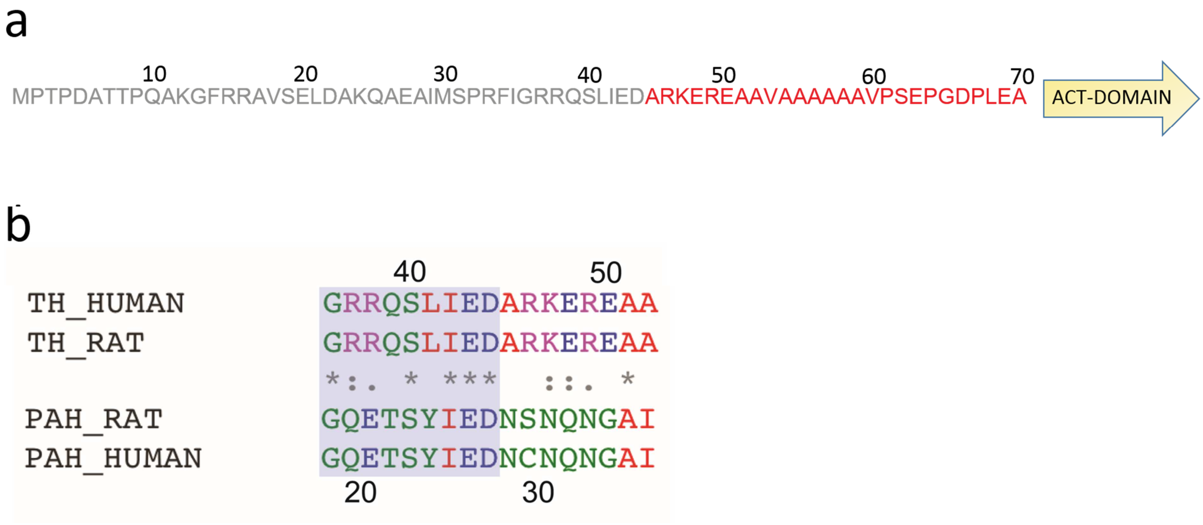

Supplementary Figure S3: The N-terminal region of TH1. (a) The N-terminal region of TH1 includes residues 1-70, for which the structure is unknown. The N-terminal tail is shown in gray and the Ala-rich segment in red (preceding the ACT-domain). (b) Sequence alignment of TH1 and human phenylalanine hydroxylase (PAH) at positions Gly36-Ala52 (for TH1), and Gly19-Ile35 (for PAH) (see main text for details).

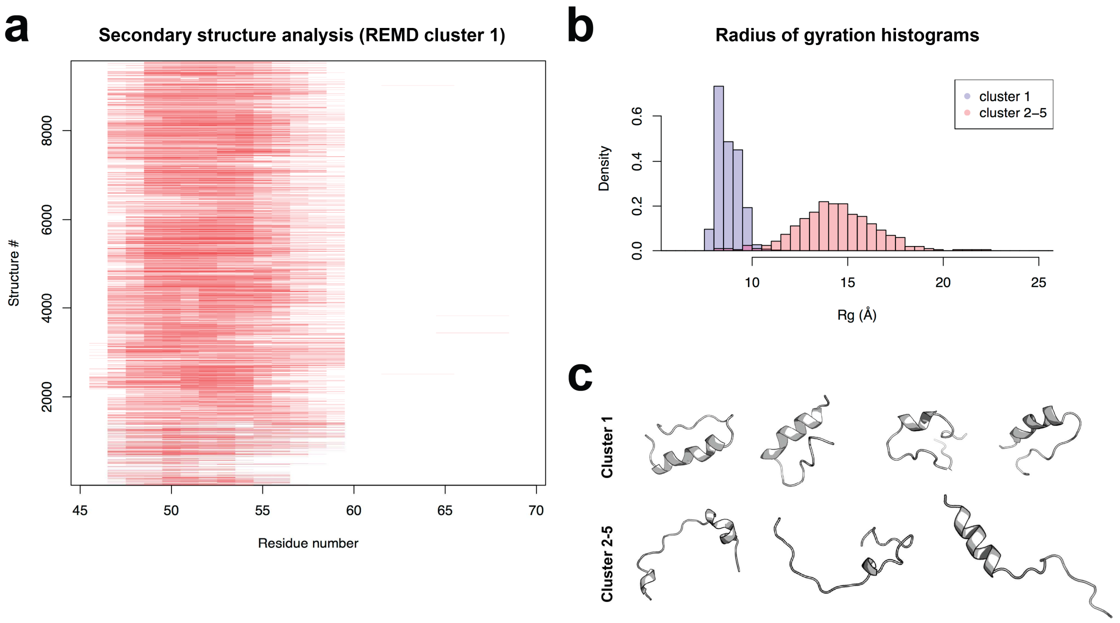

Supplementary Figure S4: Replica exchange molecular dynamics (REMD) simulations summary. (a) Schematic representation of the α-helix propensity for each of the resulting conformers in cluster 1 obtained from REMD simulations. Red colors depict presence of α-helix while white color depicts no secondary structure element. (b) Histogram of the radius of gyration for conformers in cluster 1 (blue bars) and clusters 2-5 (red bars). For comparison probability densities (so that the histogram has a total area of one) are plotted instead of frequencies. (c) Example conformers from cluster 1 (first row) and clusters 2-5 (second row).

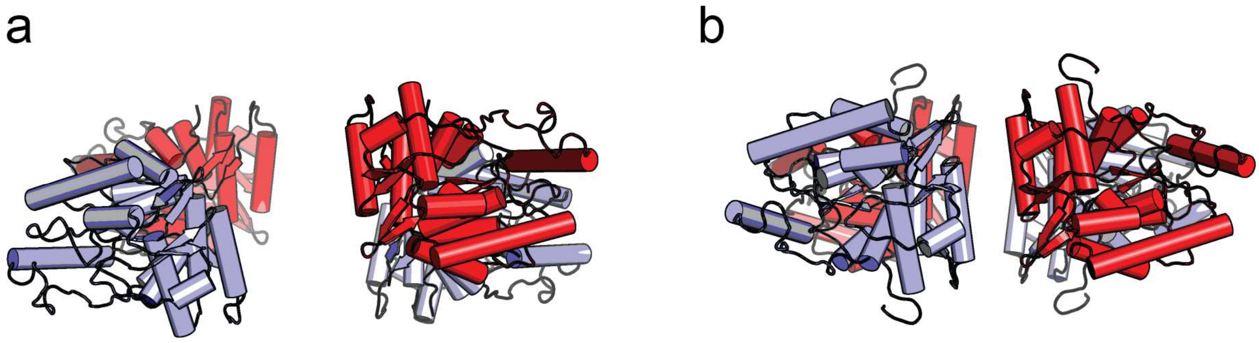

Supplementary Figure S5: Comparison of the configuration of catalytic domains in (a) the SAXS-derived hybrid model of TH1(ZZ) and (b) crystal structure of rat TH (PDB ID 1TOH). Only the catalytic domains are shown for illustration of the out-of-plane (SAXS) and in-plane (crystal) configurations.
